# A Retrospective Cohort Study on One-Year Mortality Following Emergency Laparotomy: A Tertiary Centre Experience From Western Australia

**DOI:** 10.7759/cureus.50718

**Published:** 2023-12-18

**Authors:** Mihindukulasuriya Y Pinto, Ashley O Frois, Dieter Weber

**Affiliations:** 1 Department of General Surgery, Royal Perth Hospital, Perth, AUS

**Keywords:** long-term outcome, one-year mortality, covid-19, retrospective cohort study, emergency laparotomy

## Abstract

Background

Emergency laparotomy is a common general surgical procedure associated with a high mortality and morbidity profile. While short-term outcomes following emergency laparotomy have been increasingly described, there remains a paucity of literature on long-term outcomes in Australia. We report our one-year mortality following emergency laparotomy at Royal Perth Hospital, Australia.

Methodology

A retrospective observational series of emergency laparotomies performed during 2019 and 2020 at Royal Perth Hospital was collected. The primary endpoint is the one-year mortality, and the secondary endpoints are patient demography, COVID-19 status, ASA classification, surgical category, operative indication, primary surgical pathology, procedure and surgical duration, ICU stay, post-operative destination, length of stay, 30-day mortality, and 90-day mortality. Subgroup analysis was performed for years 2019 and 2020.

Results

A total of 272 emergency laparotomies were performed during the two-year study period. The average age was 61 years (range 18- 98, SD ± 18.32). The majority of patients were in the ASA classification III (n= 134, 49.26%). The average length of patients’ stay was 14.17 days (median 10, IQR 11). Moreover, 31.98% of patients were admitted directly to the ICU following emergency laparotomy. One year mortality was 16.6%. However, a significant difference in the long-term mortality rates was observed between the two calendar years, 24.6% in 2019 and 8.66% in 2020. The one-month mortality rate was 7.33%, and the three-month mortality rate was 10.85%.

Conclusion

The one-year mortality rate observed is high and considerable and similar to experiences published elsewhere. The significant reduction in mortality during the study period warrants further investigation and may reflect improved planning and attitudes around these high-risk surgeries.

## Introduction

Emergency laparotomy (EL) is a common emergency surgical procedure that carries a high mortality and morbidity profile; the one-year outcome following EL is in the range of 15.1%-47% [1,2]. Operative Indications vary from simple and straightforward cases (e.g., the division of a single band adhesion in a small bowel obstruction) to complex and staged procedures (e.g. a staged, multi-specialty approach to a patient with ischemic bowel from a mesenteric artery thrombus).

The high associated mortalities in this EL cohort have sparked considerable academic and clinical interest in these patients. The epidemiology of ELs and their short-term outcomes were first documented in the National Emergency Laparotomy Audit (NELA) in England and Wales [3], though similar audits yielding comparable results were replicated in other healthcare regions, including The Netherlands, Denmark, and Australia. In parallel with this new understanding, improving outcomes were also reported [4,5]. These improved outcomes have been credited to numerous advances in this field, including the development and use of dedicated risk assessment tools [6-8] to guide clinical decision-making and algorithms, advocacy for high-quality goals of care discussions, early senior clinical attention, and other components that are usually incorporated into the quality improvement of care for EL patients.

While short-term outcomes following EL, including in-hospital mortality, 30-day mortality, and 90-day mortality in Australia [9-13] and worldwide [4,14-18] have been well-looked at and published, there is a paucity of literature regarding the long-term outcomes following EL. We offer this series with the aim to further understand our current practice and its long-term effect on our patients. We aim to define our current standard outcomes in one year and offer this as a point for hypothesis generation regarding the potential focus of future research.

## Materials and methods

Study population

This is a single-centre retrospective observational cohort study. Emergency laparotomies performed from 1 January 2019 to 31 December 2020 by the Department of General Surgery - Royal Perth Hospital, Western Australia, Australia, were included. This is a tertiary adult care hospital. There was no age limit for patient selection.

Inclusion criteria

All emergency laparotomies performed by the Department of General Surgery were included. Emergency laparotomies following laparoscopic or elective laparotomy and multidisciplinary laparotomy with other surgical department involvement were also included.

Exclusion criteria

Laparotomies by the Trauma Department and those conducted by specialists outside of general surgery (e.g., Urology and Gynaecology) were excluded. Relook laparotomies following an EL were also excluded. In this study, we have not included laparotomies performed for uncomplicated appendiceal or gallbladder pathology.

Data collection

Data were collected using the electronic database. Patient selection was done using the clinical code “laparotomy.” Data regarding age, gender, American Society of Anesthesiologist (ASA) classification category, primary surgical pathology, operation details, ICU admissions, and mortality were collected.

We have categorized operative indications into six broad categories: gross contamination, hemoperitoneum, necrotic bowel, bowel obstruction, and intraabdominal infection. A final category for “other” was used to designate indications not considered under the above five categories. Gross contamination is defined as a mention of “four quadrant pus or enteric content”, “pus +++”, “750 mL or more of enteric content,” or “large volume purulent fluid in the peritoneal cavity” in the operation report. Bowel obstruction is defined as a bowel obstruction without perforation or bowel ischemia. Intraabdominal infection is defined as “localized perforation or contained perforation/abscess,” “some turbid-free fluid,” or “minimal contamination or fluid in multiple areas,” as mentioned in the operation report.

Outcome

The primary outcome is one-year mortality following EL. The secondary outcomes are patient demography, COVID-19 status, ASA classification, surgical category, operative indication, primary surgical pathology, procedure and surgical duration, ICU stay, post-operative destination, length of stay, 30-day mortality, and 90-day mortality.

For the calculation of mortality, we have excluded EL in a re-admission, following an EL during our study period; our denominator was the patient count, and not the number of EL. Additionally, we have excluded the patients who lost follow-up at the one-month, three-month, and one-year marks, from the calculations of respective percentages.

Statistical analysis

The statistical analysis aimed to calculate the mortality rates and the significance level of variables between the two years. Categorical variables are presented in absolute numbers with percentages. Symmetrical continuous variables are presented in mean, range, and standard deviation (SD). Skewed continuous variables are presented in the median and interquartile range (IQR). A subgroup analysis was performed for both years.

The variables and outcomes in the two years were statistically analyzed using Welch’s T-test or Z test for two samples. The level of significance was defined as p < 0.05.

Registration

This study was approved by the institutional quality improvement committee with a waiver for ethics (Registration number 46491). This paper is reported in line with the STROCSS guidelines for observational studies [19].

## Results

Patient selection

**Figure 1:**
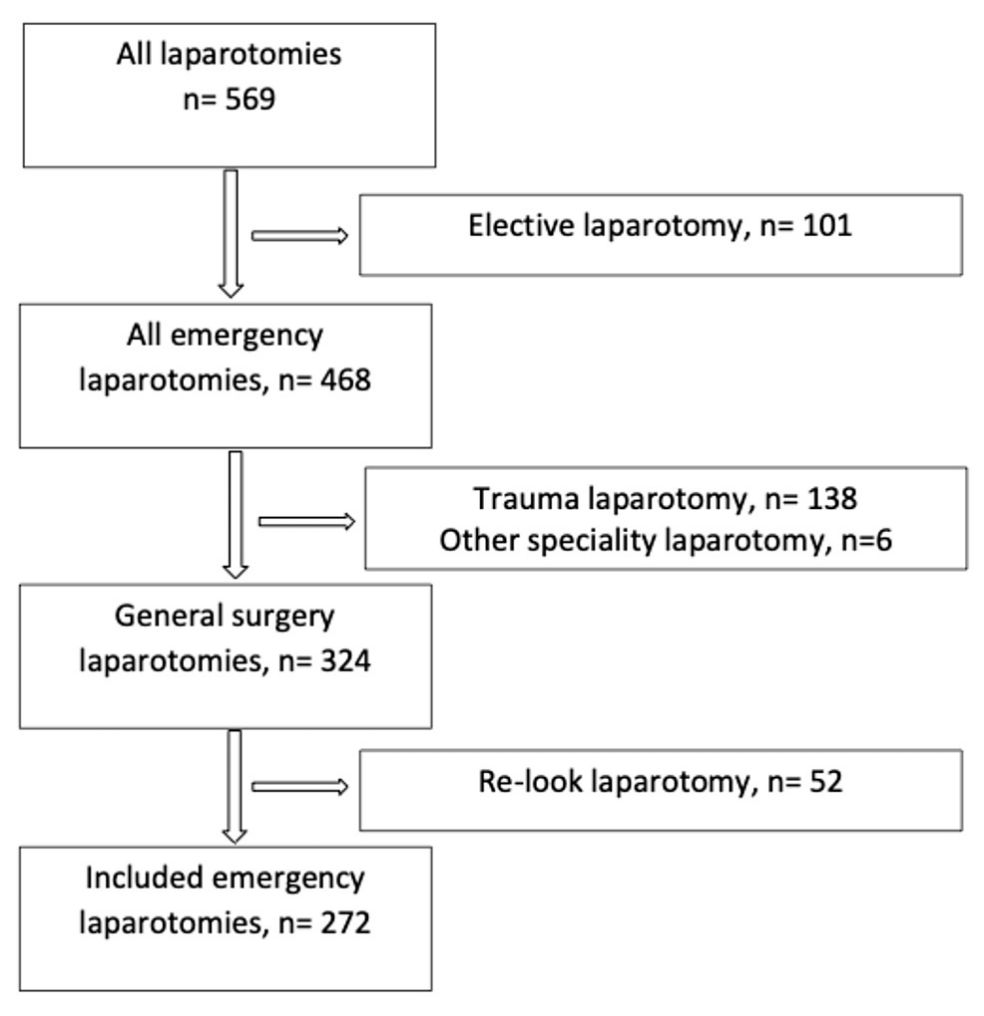
Schematic flow diagram illustrating the patient selection The data have been represented as an absolute number (n).

A total of 272 emergency general surgical laparotomies were performed across a total of 268 patients during the study period (468 emergency laparotomies were performed at Royal Perth Hospital during the study period. However, 138 trauma laparotomies, six non-general surgical emergency laparotomies, and 52 re-look laparotomies are excluded from further analysis). Four patients had re-laparotomy on re-admissions during the study period. Of the 272 ELs performed, 140 ELs were performed in 2019, and 132 ELs were performed in 2020. The overall cohort includes seven multidisciplinary laparotomies (collaboration with the surgeons from the Departments of Vascular Surgery and Urology). A schematic flow chart on patient selection is demonstrated in Figure 1.

Demography

**Figure 2:**
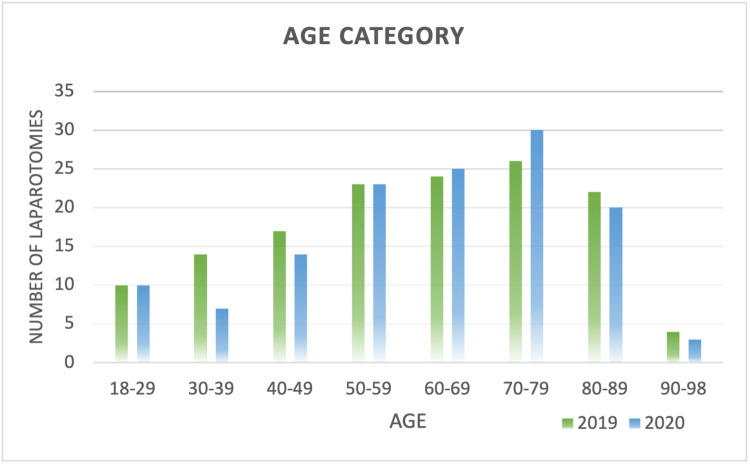
Age category and the number of laparotomies The data have been represented as an absolute number.

The mean age of a patient undergoing an EL was 61 years (range: 18-98 years, SD ± 18.32). The mean age was 61 years (SD ± 19.03) in 2019 and 62 years (SD ± 17.91) in 2020, with no significant difference between the two years (p = 0.653). The majority of patients were in the age group 70-79 (Figure 2) in both years, with 26 (18.97%) patients in 2019 and 30 (22.9%) patients in 2020. There was no significant difference in any of the age groups between those two years (Table 1). The extremes of age were not further analyzed given the small sample size. There was a slight male predominance of 55.97% (n = 150).

**Table 1:** Demography data, age categories, ASA classification, surgical category, indication, and indication category The data have been represented as an absolute number (n) and a percentage (%). p value is considered significant at p<0.05. NA = not analyzed due to small sample size, (-) = not relevant

	2019	2020	Total n (%)	p value (0.05)
Mean age (years)	61	62	61	(-)
Gender- male n (%)	75	75	150 (55.97%)	(-)
ASA classification				
I	3	9	12 (4.41%)	NA
II	19	24	43 (15.8%)	0.1
III	73	61	134 (49.26%)	0.1
IV	39	37	76 (27.94%)	0.4
V	6	1	7 (2.57%)	NA
Age groups				
18-29	10	10	20 (7.35%)	0.4
30-39	14	7	21 (7.72%)	NA
40-49	17	14	31 (11.39%)	0.3
50-59	23	23	46 (16.91%)	0.4
60-69	24	25	49 (18.01%)	0.3
70-79	26	30	56 (20.58%)	0.2
80-89	22	20	42 (15.44%)	0.4
90-98	4	3	7 (2.57%)	NA
Surgical category				
< 15 minutes	2	2	4 (1.47%)	NA
< 2 hrs	43	35	78 (28.67%)	0.44
< 6 hrs	21	31	52 (19.11%)	0.078
< 24 hrs	39	34	73 (26.83%)	0.69
< 48 hrs	34	30	64 (23.52)	0.77
unknown	1	0	1 (0.39%)	NA
Indication				
Small bowel obstruction	(-)	(-)	97 (35.66%)	(-)
Perforated colon	(-)	(-)	27 (9.92%)	(-)
Small bowel ischaemia	(-)	(-)	20 (7.37%)	(-)
Perforated small bowel	(-)	(-)	18 (6.61%)	(-)
Perforated duodenal ulcer	(-)	(-)	13 (4.77%)	(-)
Large bowel obstruction	(-)	(-)	13 (4.77%)	(-)
Indication categories				
Gross contamination	27	23	50 (18.38%)	0.3
Hemoperitoneum	12	7	19 (6.98%)	NA
Necrotic bowel	20	11	31 (11.39%)	0.06
Obstruction	51	58	109 (40.07%)	0.1
Intraabdominal infection	19	24	43 (15.8%)	0.1
Other	11	9	20 (7.37%)	NA

Pre-operative data

The majority of patients were in the ASA classification III (n = 134, 49.26%) in both years. In 2019, there were 118 patients (84.28%) in ASA classification III-V, and in 2020, there were 99 (75%). This reflected a significant difference between those two years (p = 0.02). There was no mortality in the ASA classification I or II in both years. Patients' ASA classification is summarised in Table 1.

COVID-19 status

All patients on arrival at the Emergency Department during the COVID-19 pandemic were tested for COVID-19 using a rapid antigen test. During the hospital stay, if patients manifested any symptoms related to COVID-19, they were tested again with the rapid antigen test and PCR test. Patients who had close contact prior to admission had both rapid antigen and PCR tests on admission, and they were isolated for a total of seven or 14 days since initial contact, depending on the state of emergency instructions applicable at that time. Our cohort did not have any patients test positive for COVID-19 during their index admission.

Surgical category

Urgency and the time duration for the patients to arrive in the theatres since diagnosis have been analyzed. The majority of the patients arrived in theatre within the first two hours since diagnosis (n = 78, 28.67%). Additionally, 52 (19.11%) patients got their surgery within six hours, and 73 (26.83%) patients got their surgery within the first 24 hours. Comparing the two years, there was no significant difference in the time duration for the patients to arrive in the theatres (Table 1).

Surgical indication and primary pathology

**Figure 3:**
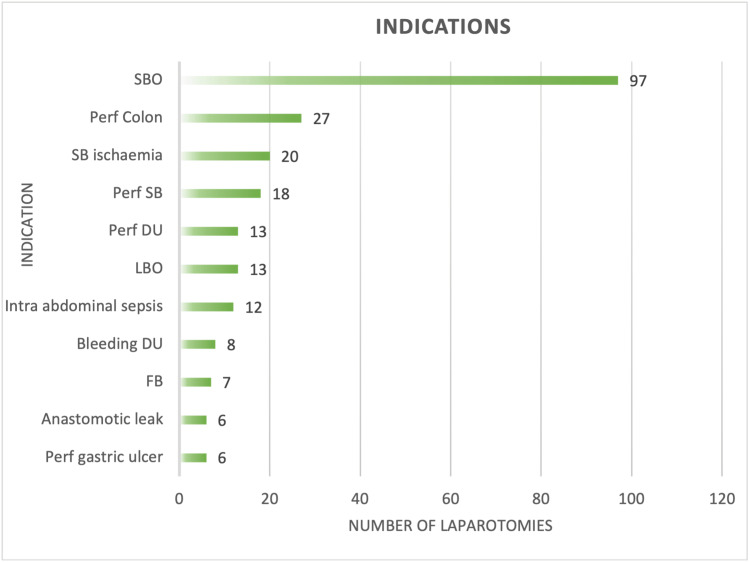
Most common indications for emergency laparotomy SBO = small bowel obstruction, Perf = perforated, SB = small bowel, DU = duodenal ulcer, LBO = large bowel obstruction, FB = foreign body The data have been represented as an absolute number.

The main indication for EL was small bowel obstruction, with a total of 97 (35.66%). This is followed by perforated colon (n = 27, 9.92%), small bowel ischemia (n = 20, 7.37%), perforated small bowel (n = 18, 6.61%), perforated duodenal ulcer (n = 13, 4.77%), and large bowel obstruction (n = 13, 4.77%) (Figure 3). Laparotomy for small bowel obstruction (n = 52) and perforated colon (n = 17) was higher in 2020 with a significant difference. Analyzing indication categories, obstructions (including both small and large bowel) were the main cause of EL, with a total of 109 (40.07%) patients. Comparing 2019 and 2020, there was no significant difference in the indication categories.

The most common procedure performed at EL was an adhesiolysis (n = 56, 20.58%). This was carried out in one of five ELs. The next most common procedure was a small bowel resection with primary anastomosis (n = 46, 16.91%), followed by a Hartmann-type procedure (n = 18, 6.61%). A total of 33 patients underwent adhesiolysis in 2020, which showed a significant increase from 2019 (p = 0.04). However, there was no significant difference in the patients who had small bowel resection with primary anastomosis in those two years (p = 0.2). Indications, indication category, and surgical category are summarised in Table 1.

Procedure duration and surgical duration

The mean procedure duration was 135.80 minutes (range: 26-497 minutes, SD: 76.75), and the mean surgical duration was 127.81 minutes (range: 19-495 minutes, SD: 75.57). Both surgical duration and procedure duration were significantly lower in 2019 (procedure duration in 2019: 125 minutes, 2020: 148 minutes, p = 0.01; surgical duration in 2019: 118 minutes, 2020: 139 minutes, p = 0.02).

Post-operative destination, length of stay, and ICU admissions

Fifty-seven (19.11%) of theatre admissions were directly from the ICU. Out of this, 34 were during 2019 and 23 during 2020, which showed a significant difference.

Following emergency laparotomy, 185 (68.01%) patients were admitted to the ward, and 87 (31.98%) to the ICU. Patients who transferred from the ICU to theatres for surgery ended up going back to the ICU other than one patient (in 2019 following adhesiolysis for small bowel obstruction) who got admitted to the ward. There were an additional 17 patients who were admitted to the ICU post-operative in 2019 and 14 in 2020.

The average length of patients’ stay was 14.17 days (median: 10, IQR: 11). This was slightly higher in 2019 with an average stay of 15.48 days (median: 12, IQR: 11.25) when compared to 12.78 days (median: 9, IQR: 9.25) in 2020.

One-year mortality outcome

**Figure 4:**
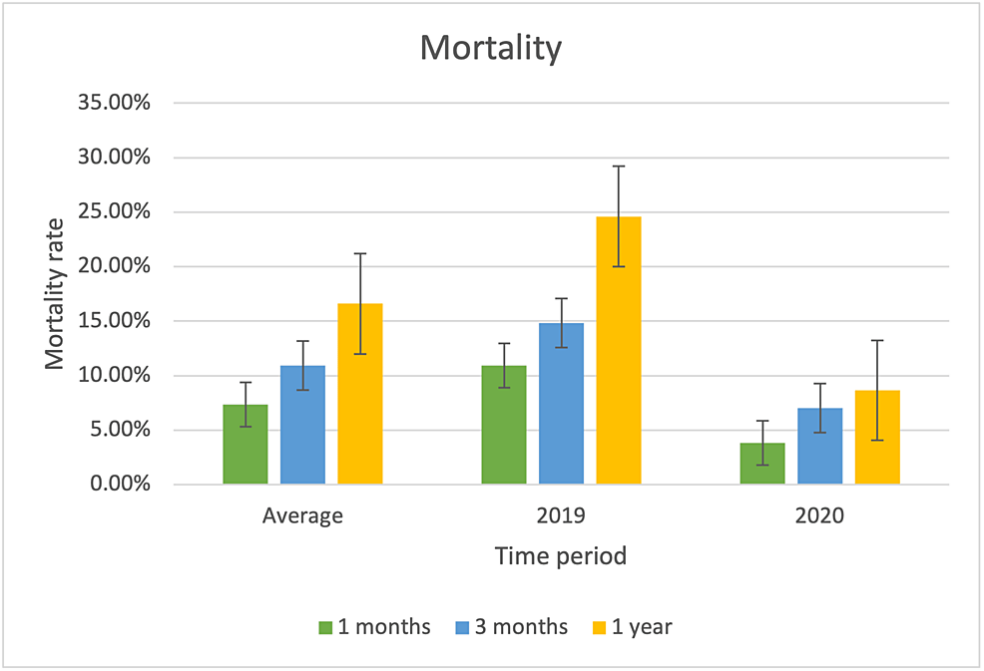
Mortality rate: one-month, three-month, and one-year mortality in 2019 and 2020 and mean value The data have been represented as a percentage.

In one year, 211 of the patients were alive. In total, this represents a one-year mortality of 16.6%. Breaking down into the calendar years, 95 patients in the 2019 cohort and 116 in the 2020 cohort were alive at this one-year timepoint. This equates to a 2019 cohort one-year mortality of 24.6% and a 2020 cohort one-year mortality of 8.66%. This reduction is statistically significant (p = 0.007) (Figure 4). All but 15 patients were able to be identified for the one-year follow-up timepoint; all uncontactable patients were from other states or internationally.

One-month and three-month mortality

One-month mortality was 7.33% (n = 19), and three-month mortality was 10.93% (n = 28). One-month mortality in 2019 was 10.85% (n = 14), and that in 2020 was 3.87% (n = 5). Three-month mortality in 2019 was 13.95% (n = 19), and that in 2020 was 7.03% (n = 9). Again, there was a significant difference in the mortality rates in one month (p = 0.03) and three months (p = 0.04). This is summarised in Figure 4.

Mortality by cause

Seventeen (40.47%) deaths were due to primary surgical pathology, and 13 (30.95%) were due to a cause unrelated to the primary surgical pathology. There was a total of eight patients (19.04%) deceased due to medical complications following the index procedure.

The majority of mortalities between three months to one year were due to directly non-related causes to the primary surgical pathology (six) and unknown causes (four). There was one death from end-stage ovarian cancer (primary surgery was for small bowel obstruction from mesenteric metastasis) and one death from urinary tract infection during chemotherapy for stage IV adenocarcinoma of ascending colon. There were four deaths from end-stage malignancies of the gastrointestinal tract (the index procedure was a complication of the malignancy).

Mortality and ASA classification

Specifically, 59.52% (n = 25) of mortality is from the ASA classification IV. This includes 16 (51.61%) patients in 2019 and nine patients (81.81%) from 2020. There were no mortalities in the ASA groups I and II. Due to the small sample size of the mortality group, we have not statistically analyzed the mortality and ASA classification in the two groups.

Mortality and the indication category

In 2019, there were 12 (38.7%) patients each in the categories of “necrotic bowel” and “obstruction.” In 2020, there were three patients (27.27%) in each of the categories “necrotic bowel,” “obstruction,” and “intrabdominal infection.” Due to a small sample size, we have not statistically analyzed the indication category and the mortality.

## Discussion

In our experience, EL procedures continue to be associated with significant mortality, though we observed a significant decline between the two calendar years reviewed. This one-year mortality rate appears comparable to international colleagues who have reported this metric. However, the diversity of the population, primary indication and surgical factors, available resources in different hospitals and theatre availability, and the expertise level of the primary operator complicates direct comparison.

Considering this fact, none of the long-term mortalities in this cohort were due to the primary procedure or a complication following, and the majority were due to a directly non-related cause from the primary pathology or the procedure. Hence, one could hypothesize that the one-year mortality rate is not related to EL outcome. Could deconditioning and change in the physiological reserve following the surgery, especially in the elderly population, had an effect on the unrelated mortalities? Accounting for the limitations of this type of study design, the conclusion remains inconclusive.

The cause of the reduction in mortality between the study years is not clear. This observation also appears to affect the short term; a similar reduction in one- and three-month mortality is also observed. There does not appear to be any significant difference in the total number of ELs and indication categories in those two years. Nonetheless, we have noticed a significant difference in the main two indications, ASA classifications III-V, commonly performed procedure, theatre admission from the ICU, and post-operative ICU admissions. ASA classifications III-V, post-operative ICU admissions, and patient arrival to theatres from the ICU were higher in the year 2019. Patients who underwent EL for small bowel obstruction and perforated colon were higher in the year 2020. Additionally, a significantly higher proportion of patients underwent adhesiolysis in 2020 compared to 2019.

Collectively, these demographic data suggest that there may have been a more physiologically ill cohort in 2019 compared with 2020. Unfortunately, separate physiological metrics at the time of diagnosis or theatre are unavailable in this study cohort. However, other factors also significantly affected and altered clinical practice during these years. During this period, clinical practice at our hospital has been profoundly affected by active recent discussions on ceilings of care/goals of care, with an increased multidisciplinary focus on delivering what that matters to the patient. This reorientation to a more patient-focused treatment strategy has intersected with a hospital-wide interest in the outcomes and key performance indicators of the binational EL audit (the Australian and New Zealand Emergency Laparotomy Audit) conducted through the Royal Australian College of Surgeons. We hypothesize that, together with the benefits of ongoing medical and surgical advances, better patient selection and more informed decision-making by the patient and clinical teams are responsible for this significantly reduced mortality.

To further complicate the comparison between 2019 and 2020, the COVID-19 pandemic drastically altered our patient population’s behavior and our clinical practice. While there were no COVID-19 patients in our 2020 cohort, there was a significant difference in the mean procedure time and mean surgical time, which was higher in the pandemic year. This could be due to the introduction of mandatory full personal protective equipment protocols and possible theatre sterilizing before and after the procedure in the pandemic year, as well as changes in seniority of staffing of operating theatre personnel/grade of surgeon operating.

This retrospective case series is limited by the usual definitional and methodological issues inherent in this study type. We rely on the data entered retrospectively on the electronic database and are dependent on the accuracy of our clinical coding. The numbers of various categories are small and may contribute to possible type II errors.

## Conclusions

In conclusion, our tertiary hospital clinical experience suggests that current emergency general surgery practice continues to attend a sick cohort of patients requiring ELs for a variety of pathologies and with the need for a range of operations. These ELs are associated with measurable mortality, both in the short, as well as in the longer term. We hope this experience can assist in guiding further research such that future surgeons and patients may make better-informed decisions about their treatment goals and expected outcomes.
